# Progress in Cancer

**Published:** 1902-09-13

**Authors:** 


### PROGRESS IN CANCER.

While in England, Sir Mitchell Banks[1] gives it as his opinion that cancer is affecting young persons more frequently than it used to do. Dr. Tatham[2] finds that this increased incidence is affecting males far more than females. His figures, contrasting the ten years 1861 to 1870 with 1891–1900, show that the incidence among males has increased 147 per cent., while only 74 per cent. among females. The same general increase in the prevalence of this fell disease is to be found in the records of the Melbourne Hospital, as adduced by Professor Allen[3] for the last 25 years. During the first five years the deaths from cancer were 6·8 per cent.; in the second five years these were 6·9 per cent., with an increasing ratio till the fifth quintennium shows 10·4 per cent. deaths. Even allowing for increase of population, the death-rate has risen from 2·75 per cent. to 5·72 per cent. in the last 30 years, and in males the cancer rate has more than doubled, 2·65 to 5·91, while in females it has less than doubled, 2·86 to 5·5. As a contrast with this must be placed the striking increase in the numbers of old persons in proportion to the whole population; the old men having nearly doubled, and the old women have more than doubled. He does not, like Banks, consider that cancer is increasing among the young. Sarcoma in males over 25 is slightly decreasing, while in females it is slightly increasing. While below 43 there is no definite increase, above 45 there is a progressive increase. From 45 to 55 the increase is 42 per cent.; from 55 to 65 it is 86 per cent.; from 65 to 75 it is more than doubled, and from 75 upwards more than trebled. While the male death-rate has increased much more rapidly than the female below 75, above this age the female rate has increased $4\frac{1}{4}$ times to the male $2\frac{1}{2}$ times. In certain positions there is a definite increase in cancer incidence—for the stomach it has doubled in both sexes; for the liver in men it has trebled, but it is five times as much in women. In the rectum it has increased three times in women, but ten times in men. Generally cancer of the face and lip is less frequent, cancer of the breast slightly less frequent, but cancer of the uterus slightly increased. Contrary to the statement made by McFadyean in the cancer number of the *Practitioner*, that while cancer was found in the horse, dog, cat, and sheep it was very rare in the pig. Gaylord reports that Dr. Zink, who has exceptional opportunities of investigating the subject, has found four cases of malignant tumour in swine in four months out of about 2,000 animals. It is not to be wondered at that, with the many investigators at work upon the subject, their expressed views should differ so widely from each other. Payne[5] considers that the explanations of epithelial cancers and sarcomata may be different. He looks at the subject from a natural history point of view. If we are to regard cancer as a specific disease, we should be able to find evidence of (1) an initial lesion, secondary lesions and of general intoxication; (2) communication of the disease from one animal to another, whether of the same species or not, by contagion, inoculation or any experimental method; (3) evidence of a specific, local or geographical distribution, such as has been demonstrated in malaria for example. Now cancer originally is a local process with secondary seats set up by the conveyance of something from the primary seat; also there is a general intoxication. Immunity is conferred against attacks of the same organism, multiple primary cancers being almost unknown. For the most part cancer would appear to be not inocculable, though in some instances there would seem to be exceptions to the rule where husband and wife are both affected. He says that 70 per cent. of cancers begin on the external or internal surface of the body, using the word "surface" in its broadest sense to include glandular involutions, etc. The incidence of cancer upon these may be regarded in the light of direct infection. With regard to the sebaceous glands—and the breast may be regarded as a specialised sebaceous gland—it is possible that the cancerous poison or microbes are excreted through these paths, and that all goes well until the microbes happen to become arrested in their transit and then grow *in situ*. In a healthy woman before the climacteric the periodic discharge of menstrual blood may carry off the infection; when this stops the germs may develop *in situ*. In old days discharging sores were popularly supposed to be incompatible with the development of carcinoma. The increased incidence of cancer has been noticed since periodic phlebotomy, as a regular practice, has been stopped. With regard to the geographical distribution there is no doubt that cancer is more prevalent in some parts of the world than in others. Even among the same communities, some parts are more prone to it than others, even though the general death-rate may be lower than the average. It may be that some marshy districts are infected soils, or that they favour the presence of certain insects. This, too, will account for marshy lands where cancer is not prevalent. There is nothing to forbid the supposition then, though clear evidence is wanting, that the existence of cancer depends in certain localities upon the presence there of some form of animal or vegetable life. With relation to the nature of the supposed parasite Dr. Galloway,[6] after examining coccidia and their phenomena, finds it difficult to believe that the cause of cancer is protozoal in origin. The fungoid origin is hard to accept also, for blastomycetic dermatitis and blastomycosis are definite affections, really granulomata, and it is difficult to believe that they could form the complex carcinomata and sarcomata. And, secondly, it has been observed that cancers have become infected by hyphæ-producing fungi. If such a tumour were examined bacteriologically the fungus would grow, and hence a wrong impression would be acquired. Beatson[7] looks to the question of the internal secretion of the cells of testes and ovary for an explanation of the cause of cancer rather than to any parasite; an influence predominating over the proliferation of cells in the body. This becomes most apparent when for any reason a cell is incapable of carrying out its highest function. Thus after long-continued irritation there is a loss of differentiation and lowered functional vitality of the cell and therefore a greater proliferative power. Again, the tissues in which the senile changes occur early, as, for instance, the breast or uterus, show especial liability to cancer. When age comes on cancer is more prone to manifest itself. The question of heredity is a question really of inherent vitality in the tissues. The idea of cancer houses is to be explained by the environment which has a general effect in lessening the functional activity of the cells. During pregnancy the body cells are stimulated to produce a larger amount than usual of the "body fluid," which makes cancers flourish, and hence cancers grow with greater rapidity during pregnancy. If long after the removal of a breast recurrence takes place *in situ*, the explanation may be that a part of the undeteriorated breast-tissue had been left behind, and later the same influences which had been at work to produce the original tumour have at length produced the nodule in the scar. To recapitulate (1) the cancer process can only develop in tissues that have special proliferating power, either natural as in Cohnheim's "rests," or acquired in one of many ways; (2) that the actual exciting cause of the cell proliferation is a secretion from all the tissues of the body and possessed of fructifying powers; (3) that a mutual relationship exists between the character and amount of the secretion and the testes and ovaries; (4) that another factor in connection with it is the inherent vitality of the tissues themselves. According to Marmaduke Shield[8] the fact that no recurrence takes place after an operation for cancer for so long periods as 11 to 12 years is not due to the skill of the surgeon, but is due "to an undetermined factor in some individuals which militates against the return of the disease." D'Arcy Power[9] remarks on the power flies possess of carrying the germs of disease, enteric, cholera, tuberculosis, etc., and with this in view he has examined microscopically flies from a cancer house, but can as yet find no difference from other flies. On investigation and inquiry he finds that there are two kinds of house-fly, viz., the common house-fly which has no power of penetrating the human skin, and as Dr. Lowne has pointed out that the "stomoxys calcitrans," which besides having the distinction of holding its head erect, and having white interrupted bands on the body, has the power of biting, and this function it exercises usually in the late autumn. These insects are found in towns, but especially where cattle abound and in low-lying districts. With these facts before him he wishes to investigate whether the stomoxys is the commonest fly about cancer houses; whether there can be obtained any history of fly-bites in cancer patients; and to investigate the microscopical appearances of these insects, to find out any "cancer bodies," or any possible indication of the fly being an intermediate host. From a study of Deciduoma malignum, Adami[10] states that it is a syncytioma or chorioepithelioma malignum, and that no other view is tenable. From this he deduces (1) that there exists one form of tumour of highly malignant type, in which the infiltrating cells are not those of the organism itself, but are derived from another organism. (2) The infiltrative and invasive properties are not a new acquirement, but are an exaltation of properties normally possessed by them. There being an interaction between the fœtal and maternal cells, the invasion of the cells being strictly limited by the interaction of the placental site. (3) The development of this syncytioma malignum is due then to either an increase of the invasive properties of the syncytial cells, or to a decrease in the defence of the maternal cells. (4) To produce this must be the part played by microbes, if such exist. (5) Positive proof of such parasites is wanting, and it is difficult to conceive of such results being caused by specific microparasites. (6) Admitting that microparasites have the power of producing cell proliferation, it must also be allowed that other external agencies have this power too, and thus the microparasites at most might be one of the causes of cell-growth. (7) The prevalent conception of cancer parasites existing within the cancer cells involves the idea of malignant growths being the products of parasites acting within parasites: the syncytioma cells themselves act as parasites. All he can see in the microbic view is a possible addition to the causes of pre-cancerous irritation. It is of the utmost advantage to obtain information relative to cancer from whatever source available, and particularly so when other scientists than medical men join their evidence with ours. A paper of the highest interest appears in the *Lancet*, June 21st, from the pen of Dr. J. Beard,[11] who opens up the question of the ætiology of cancer from the embryological side. He considers that to look for the cause of cancer in some unicellular parasite is futile. It is not in the nature of parasitic maladies to lead to a cellular increase such as is characteristic of cancer. It is this unrestricted cellular increase and multiplication which impresses the embryologist. He would first have us consider that the "hen's producing an egg and the egg a new hen" is not the true cycle of development. It is more complex than that. There is an asexual generation unrecognised in this cycle, the amended cycle being: "Egg—larva (phorozoon, or bearing animal, or asexual generation) —primitive germ cell—primary germ cells—secondary germ cells—gametes, eggs or spermo-fertilised egg. The embryo does not appear in this cycle, it arises from one of the primary germ cells whose number is always a definite one of a geometrical series of 2*n*th power. The remaining germ cells enter the embryonic body to form its sexual products. The four important items in the cycle are:—(1) The gametes—egg or sperm by whose union a new cycle is initiated; (2) the first product of the zygote, the phorozoon, larva, or asexual generation; (3) the primary germ cells destined for future generations, and (4) the embryo or sexual generation. Every germ cell has the potentiality of becoming an embryo, but its doing so limits its power of growth and increase of life. The "phorozoon has indefinite unrestricted powers of growth in an apical fashion." If this apical growth, as it commonly is in many animals, is only in one direction, and if this gets cut off from the organism it leads to the conversion into a primitive germ cell and the origin of primary germ cells from it, and the further development of a primary germ cell is an embryo, no longer a larva. The primary germ cells being 2*n*th in number the greatest number of primary germ cells left in any embryo must necessarily be 2*n*th—1. Now it never happens that in the organism all these primary germ cells reach the germinal ridge—they may remain in unusual places and many degenerate. The vagrant germ cells vary for the species, but are to be found in positions such as the skin, body cavity, rectum, kidney, gill region, etc. The "embryomas" of Wilms are to be regarded as developments of these primary germ cells: but the cells are to be found where embryomas are never encountered, and in nearly every embryo they are to be found in the neighbourhood of the pylorus and the yolk-sac and the rectal epithelium. Now that these are just the regions where cancers are found is suggestive of the possibility of their development from these cells. The development may be anything short of complete twin embryo production, and that the vagrant germ cell omits or skips the development of an embryo is accounted for by the much later period at which the cell development occurs: it takes on a different portion of the life cycle; the abnormal situations and conditions determining the parthenogenesis or abnormal formation of a larva. The whole process is in consequence of the larval period being passed as a parasitic stage in the sexual form. A placentoma can be explained by the fact that the embryo has become aborted or has died prior to the suppression of the larva, the latter, the chorion, may go on growing indefinitely and produce the phenomenon. The writer finds in this the complete and satisfactory cause of cancers of whatever form, and sums up by saying that "the vagrant primary germ cell is the seed, while its fruit, sometimes represented by an embryoma, may on occasion take the form of a carcinoma.

[1] Clin. Jour., May 28. [2] Clin. Jour., May 14. [3] Australasian Med. Gaz., April 21. [4] Clin. Jour., June 11. [5] Clin. Jour., July 2. [6] Clin. Jour., May 14. [7] Clin. Jour., May 21. [8] Clin. Jour., June 4. [9] Clin. Jour., June 11. [10] Lancet, April 5. [11] Lancet, June 21.

